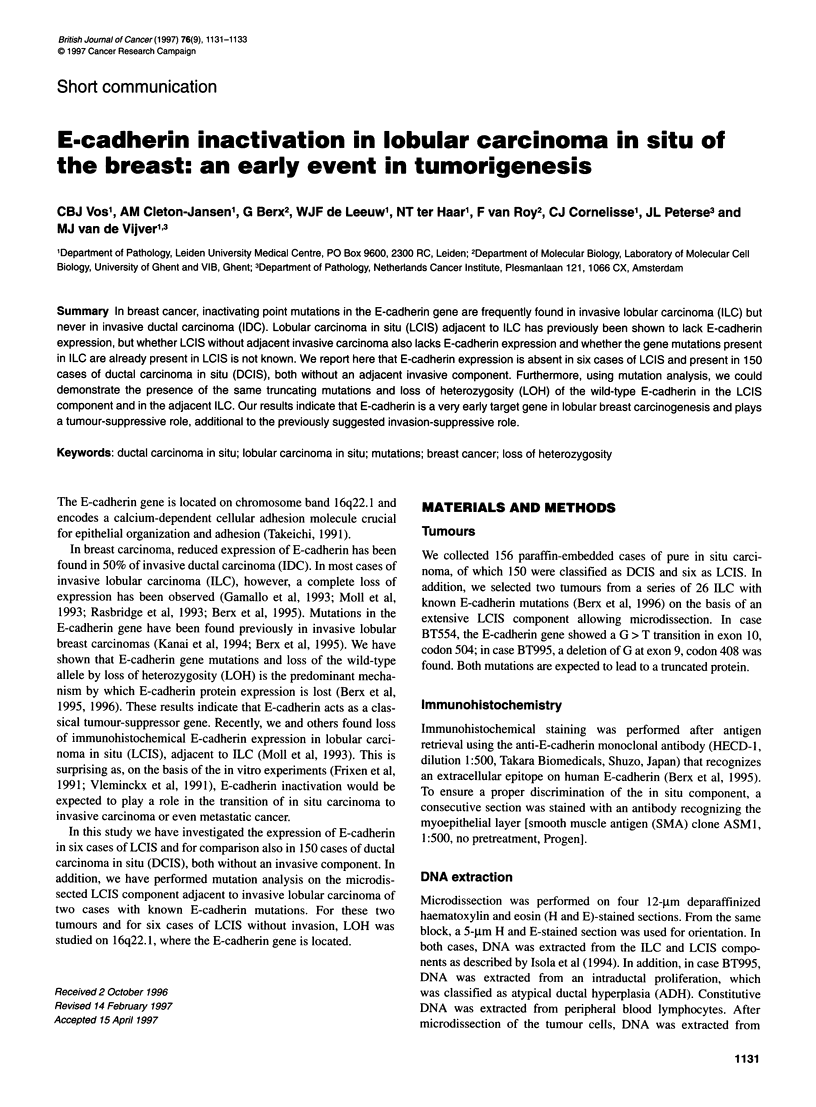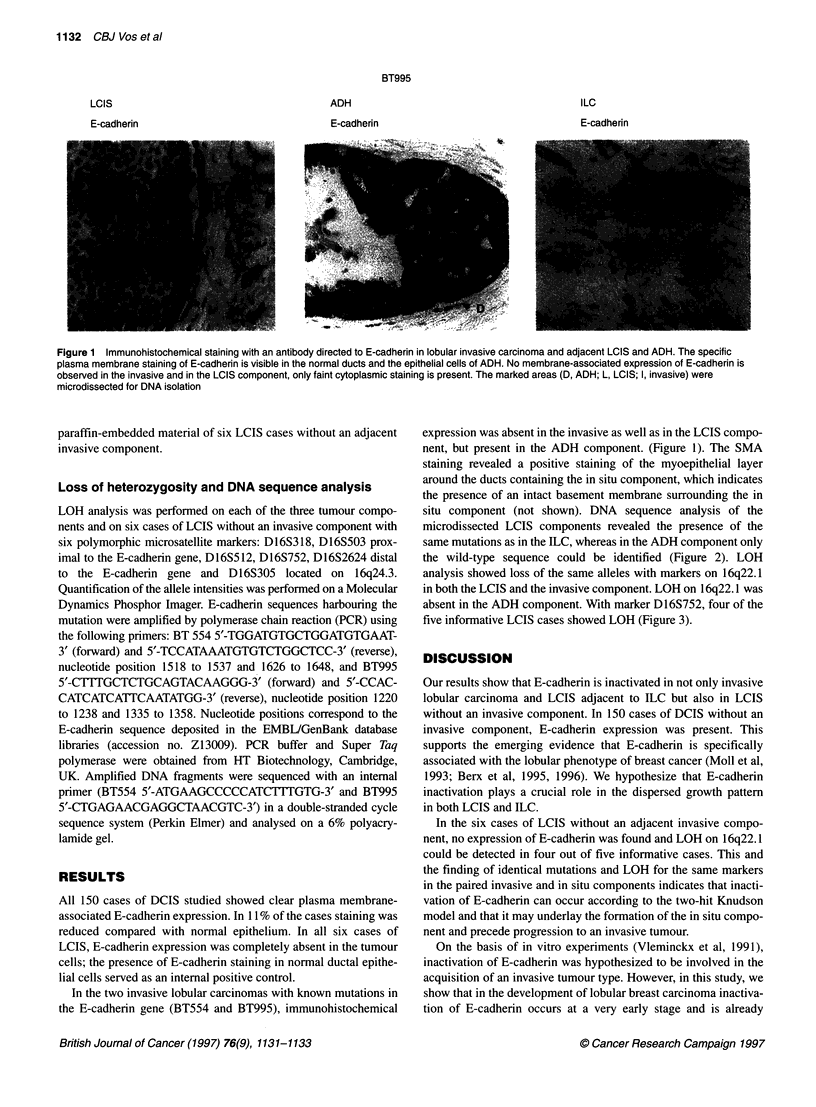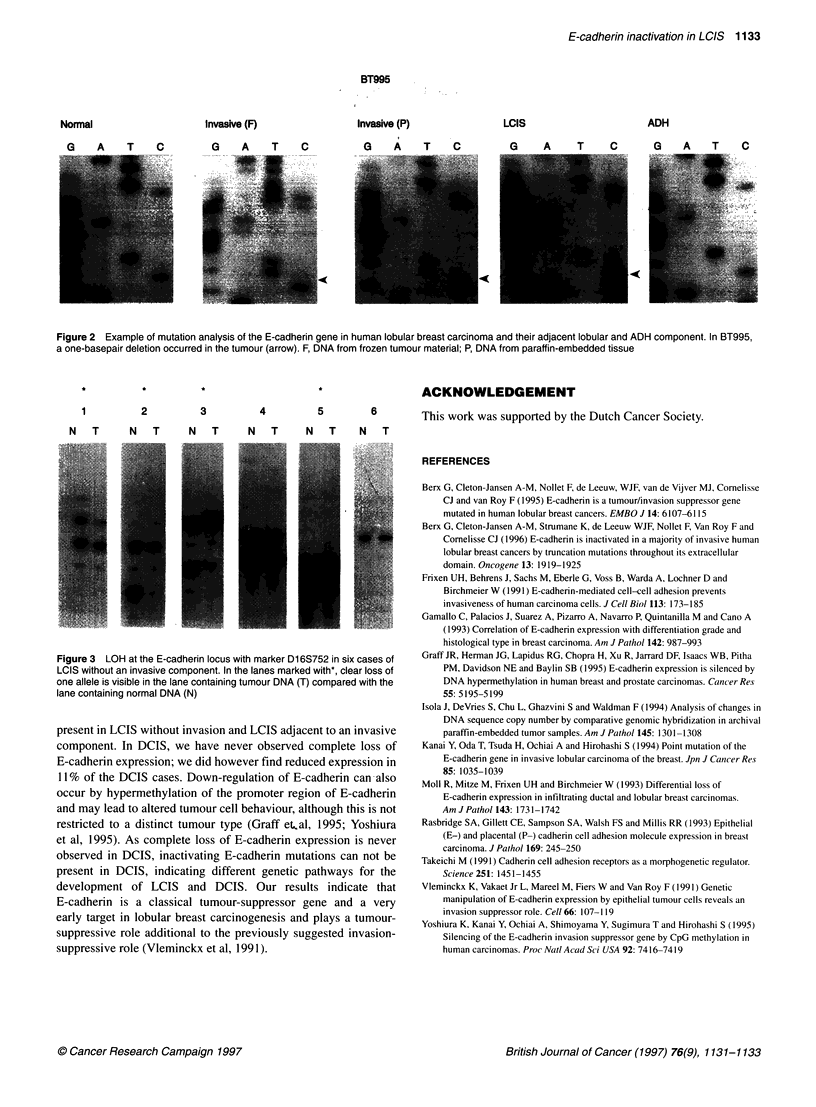# E-cadherin inactivation in lobular carcinoma in situ of the breast: an early event in tumorigenesis.

**DOI:** 10.1038/bjc.1997.523

**Published:** 1997

**Authors:** C. B. Vos, A. M. Cleton-Jansen, G. Berx, W. J. de Leeuw, N. T. ter Haar, F. van Roy, C. J. Cornelisse, J. L. Peterse, M. J. van de Vijver

**Affiliations:** Department of Pathology, Leiden University Medical Centre.

## Abstract

**Images:**


British Journal of Cancer (1997) 76(9), 1131–1133
© 1997 Cancer Research Campaign

Short communication

### E-cadherin inactivation in lobular carcinoma in situ of the breast: an early event in tumorigenesis

**CBJ Vos[1], AM Cleton-Jansen[1], G Berx[2], WJF de Leeuw[1], NT ter Haar[1], F van Roy[2], CJ Cornelisse[1], JL Peterse[3] and MJ van de Vijver[1,3]**

[1]Department of Pathology, Leiden University Medical Centre, PO Box 9600, 2300 RC, Leiden; [2]Department of Molecular Biology, Laboratory of Molecular Cell Biology, University of Ghent and VIB, Ghent; [3]Department of Pathology, Netherlands Cancer Institute, Plesmanlaan 121, 1066 CX, Amsterdam

**Summary** In breast cancer, inactivating point mutations in the E-cadherin gene are frequently found in invasive lobular carcinoma (ILC) but never in invasive ductal carcinoma (IDC). Lobular carcinoma in situ (LCIS) adjacent to ILC has previously been shown to lack E-cadherin expression, but whether LCIS without adjacent invasive carcinoma also lacks E-cadherin expression and whether the gene mutations present in ILC are already present in LCIS is not known. We report here that E-cadherin expression is absent in six cases of LCIS and present in 150 cases of ductal carcinoma in situ (DCIS), both without an adjacent invasive component. Furthermore, using mutation analysis, we could demonstrate the presence of the same truncating mutations and loss of heterozygosity (LOH) of the wild-type E-cadherin in the LCIS component and in the adjacent ILC. Our results indicate that E-cadherin is a very early target gene in lobular breast carcinogenesis and plays a tumour-suppressive role, additional to the previously suggested invasion-suppressive role.

**Keywords**: ductal carcinoma in situ; lobular carcinoma in situ; mutations; breast cancer; loss of heterozygosity

The E-cadherin gene is located on chromosome band 16q22.1 and encodes a calcium-dependent cellular adhesion molecule crucial for epithelial organization and adhesion (Takeichi, 1991).

In breast carcinoma, reduced expression of E-cadherin has been found in 50% of invasive ductal carcinoma (IDC). In most cases of invasive lobular carcinoma (ILC), however, a complete loss of expression has been observed (Gamallo et al, 1993; Moll et al, 1993; Rasbridge et al, 1993; Berx et al, 1995). Mutations in the E-cadherin gene have been found previously in invasive lobular breast carcinomas (Kanai et al, 1994; Berx et al, 1995). We have shown that E-cadherin gene mutations and loss of the wild-type allele by loss of heterozygosity (LOH) is the predominant mechanism by which E-cadherin protein expression is lost (Berx et al, 1995, 1996). These results indicate that E-cadherin acts as a classical tumour-suppressor gene. Recently, we and others found loss of immunohistochemical E-cadherin expression in lobular carcinoma in situ (LCIS), adjacent to ILC (Moll et al, 1993). This is surprising as, on the basis of the in vitro experiments (Frixen et al, 1991; Vleminckx et al, 1991), E-cadherin inactivation would be expected to play a role in the transition of in situ carcinoma to invasive carcinoma or even metastatic cancer.

In this study we have investigated the expression of E-cadherin in six cases of LCIS and for comparison also in 150 cases of ductal carcinoma in situ (DCIS), both without an invasive component. In addition, we have performed mutation analysis on the microdissected LCIS component adjacent to invasive lobular carcinoma of two cases with known E-cadherin mutations. For these two tumours and for six cases of LCIS without invasion, LOH was studied on 16q22.1, where the E-cadherin gene is located.

*Received 2 October 1996*
*Revised 14 February 1997*
*Accepted 15 April 1997*

### MATERIALS AND METHODS

### Tumours

We collected 156 paraffin-embedded cases of pure in situ carcinoma, of which 150 were classified as DCIS and six as LCIS. In addition, we selected two tumours from a series of 26 ILC with known E-cadherin mutations (Berx et al, 1996) on the basis of an extensive LCIS component allowing microdissection. In case BT554, the E-cadherin gene showed a G > T transition in exon 10, codon 504; in case BT995, a deletion of G at exon 9, codon 408 was found. Both mutations are expected to lead to a truncated protein.

### Immunohistochemistry

Immunohistochemical staining was performed after antigen retrieval using the anti-E-cadherin monoclonal antibody (HECD-1, dilution 1:500, Takara Biomedicals, Shuzo, Japan) that recognizes an extracellular epitope on human E-cadherin (Berx et al, 1995). To ensure a proper discrimination of the in situ component, a consecutive section was stained with an antibody recognizing the myoepithelial layer [smooth muscle antigen (SMA) clone ASM1, 1:500, no pretreatment, Progen].

### DNA extraction

Microdissection was performed on four 12-μm deparaffinized haematoxylin and eosin (H and E)-stained sections. From the same block, a 5-μm H and E-stained section was used for orientation. In both cases, DNA was extracted from the ILC and LCIS components as described by Isola et al (1994). In addition, in case BT995, DNA was extracted from an intraductal proliferation, which was classified as atypical ductal hyperplasia (ADH). Constitutive DNA was extracted from peripheral blood lymphocytes. After microdissection of the tumour cells, DNA was extracted from

**Figure 1** Immunohistochemical staining with an antibody directed to E-cadherin in lobular invasive carcinoma and adjacent LCIS and ADH. The specific plasma membrane staining of E-cadherin is visible in the normal ducts and the epithelial cells of ADH. No membrane-associated expression of E-cadherin is observed in the invasive and in the LCIS component, only faint cytoplasmic staining is present. The marked areas (D, ADH; L, LCIS; I, invasive) were microdissected for DNA isolation paraffin-embedded material of six LCIS cases without an adjacent invasive component.

### Loss of heterozygosity and DNA sequence analysis

LOH analysis was performed on each of the three tumour components and on six cases of LCIS without an invasive component with six polymorphic microsatellite markers: D16S318, D16S503 proximal to the E-cadherin gene, D16S512, D16S752, D16S2624 distal to the E-cadherin gene and D16S305 located on 16q24.3. Quantification of the allele intensities was performed on a Molecular Dynamics Phosphor Imager. E-cadherin sequences harbouring the mutation were amplified by polymerase chain reaction (PCR) using the following primers: BT 554 5′-TGGATGTGCTGGATGTGAAT-3′ (forward) and 5′-TCCATAAATGTGTCTGGCTCC-3′ (reverse), nucleotide position 1518 to 1537 and 1626 to 1648, and BT995 5′-CTTTGCTCTGCAGTACAAGGG-3′ (forward) and 5′-CCACCATCATCATTCAATATGG-3′ (reverse), nucleotide position 1220 to 1238 and 1335 to 1358. Nucleotide positions correspond to the E-cadherin sequence deposited in the EMBL/GenBank database libraries (accession no. Z13009). PCR buffer and Super *Taq* polymerase were obtained from HT Biotechnology, Cambridge, UK. Amplified DNA fragments were sequenced with an internal primer (BT554 5′-ATGAAGCCCCCATCTTTGTG-3′ and BT995 5′-CTGAGAACGAGGCTAACGTC-3′) in a double-stranded cycle sequence system (Perkin Elmer) and analysed on a 6% polyacrylamide gel.

### RESULTS

All 150 cases of DCIS studied showed clear plasma membrane-associated E-cadherin expression. In 11% of the cases staining was reduced compared with normal epithelium. In all six cases of LCIS, E-cadherin expression was completely absent in the tumour cells; the presence of E-cadherin staining in normal ductal epithelial cells served as an internal positive control.

In the two invasive lobular carcinomas with known mutations in the E-cadherin gene (BT554 and BT995), immunohistochemical expression was absent in the invasive as well as in the LCIS component, but present in the ADH component. (Figure 1). The SMA staining revealed a positive staining of the myoepithelial layer around the ducts containing the in situ component, which indicates the presence of an intact basement membrane surrounding the in situ component (not shown). DNA sequence analysis of the microdissected LCIS components revealed the presence of the same mutations as in the ILC, whereas in the ADH component only the wild-type sequence could be identified (Figure 2). LOH analysis showed loss of the same alleles with markers on 16q22.1 in both the LCIS and the invasive component. LOH on 16q22.1 was absent in the ADH component. With marker D16S752, four of the five informative LCIS cases showed LOH (Figure 3).

### DISCUSSION

Our results show that E-cadherin is inactivated in not only invasive lobular carcinoma and LCIS adjacent to ILC but also in LCIS without an invasive component. In 150 cases of DCIS without an invasive component, E-cadherin expression was present. This supports the emerging evidence that E-cadherin is specifically associated with the lobular phenotype of breast cancer (Moll et al, 1993; Berx et al, 1995, 1996). We hypothesize that E-cadherin inactivation plays a crucial role in the dispersed growth pattern in both LCIS and ILC.

In the six cases of LCIS without an adjacent invasive component, no expression of E-cadherin was found and LOH on 16q22.1 could be detected in four out of five informative cases. This and the finding of identical mutations and LOH for the same markers in the paired invasive and in situ components indicates that inactivation of E-cadherin can occur according to the two-hit Knudson model and that it may underlay the formation of the in situ component and precede progression to an invasive tumour.

On the basis of in vitro experiments (Vleminckx et al, 1991), inactivation of E-cadherin was hypothesized to be involved in the acquisition of an invasive tumour type. However, in this study, we show that in the development of lobular breast carcinoma inactivation of E-cadherin occurs at a very early stage and is already

*© Cancer Research Campaign 1997*

**Figure 2** Example of mutation analysis of the E-cadherin gene in human lobular breast carcinoma and their adjacent lobular and ADH component. In BT995, a one-basepair deletion occurred in the tumour (arrow). F, DNA from frozen tumour material; P, DNA from paraffin-embedded tissue

**Figure 3** LOH at the E-cadherin locus with marker D16S752 in six cases of LCIS without an invasive component. In the lanes marked with*, clear loss of one allele is visible in the lane containing tumour DNA (T) compared with the lane containing normal DNA (N)

present in LCIS without invasion and LCIS adjacent to an invasive component. In DCIS, we have never observed complete loss of E-cadherin expression; we did however find reduced expression in 11% of the DCIS cases. Down-regulation of E-cadherin can also occur by hypermethylation of the promoter region of E-cadherin and may lead to altered tumour cell behaviour, although this is not restricted to a distinct tumour type (Graff et al, 1995; Yoshiura et al, 1995). As complete loss of E-cadherin expression is never observed in DCIS, inactivating E-cadherin mutations can not be present in DCIS, indicating different genetic pathways for the development of LCIS and DCIS. Our results indicate that E-cadherin is a classical tumour-suppressor gene and a very early target in lobular breast carcinogenesis and plays a tumour-suppressive role additional to the previously suggested invasion-suppressive role (Vleminckx et al, 1991).

### ACKNOWLEDGEMENT

This work was supported by the Dutch Cancer Society.

### REFERENCES

Berx G, Cleton-Jansen A-M, Nollet F, de Leeuw, WJF, van de Vijver MJ, Cornelisse CJ and van Roy F (1995) E-cadherin is a tumour/invasion suppressor gene mutated in human lobular breast cancers. *EMBO J* **14**: 6107–6115

Berx G, Cleton-Jansen A-M, Strumane K, de Leeuw WJF, Nollet F, Van Roy F and Cornelisse CJ (1996) E-cadherin is inactivated in a majority of invasive human lobular breast cancers by truncation mutations throughout its extracellular domain. *Oncogene* **13**: 1919–1925

Frixen UH, Behrens J, Sachs M, Eberle G, Voss B, Warda A, Lochner D and Birchmeier W (1991) E-cadherin-mediated cell–cell adhesion prevents invasiveness of human carcinoma cells. *J Cell Biol* **113**: 173–185

Gamallo C, Palacios J, Suarez A, Pizarro A, Navarro P, Quintanilla M and Cano A (1993) Correlation of E-cadherin expression with differentiation grade and histological type in breast carcinoma. *Am J Pathol* **142**: 987–993

Graff JR, Herman JG, Lapidus RG, Chopra H, Xu R, Jarrard DF, Isaacs WB, Pitha PM, Davidson NE and Baylin SB (1995) E-cadherin expression is silenced by DNA hypermethylation in human breast and prostate carcinomas. *Cancer Res* **55**: 5195–5199

Isola J, DeVries S, Chu L, Ghazvini S and Waldman F (1994) Analysis of changes in DNA sequence copy number by comparative genomic hybridization in archival paraffin-embedded tumor samples. *Am J Pathol* **145**: 1301–1308

Kanai Y, Oda T, Tsuda H, Ochiai A and Hirohashi S (1994) Point mutation of the E-cadherin gene in invasive lobular carcinoma of the breast. *Jpn J Cancer Res* **85**: 1035–1039

Moll R, Mitze M, Frixen UH and Birchmeier W (1993) Differential loss of E-cadherin expression in infiltrating ductal and lobular breast carcinomas. *Am J Pathol* **143**: 1731–1742

Rasbridge SA, Gillett CE, Sampson SA, Walsh FS and Millis RR (1993) Epithelial (E–) and placental (P–) cadherin cell adhesion molecule expression in breast carcinoma. *J Pathol* **169**: 245–250

Takeichi M (1991) Cadherin cell adhesion receptors as a morphogenetic regulator. *Science* **251**: 1451–1455

Vleminckx K, Vakaet Jr L, Mareel M, Fiers W and Van Roy F (1991) Genetic manipulation of E-cadherin expression by epithelial tumour cells reveals an invasion suppressor role. *Cell* **66**: 107–119

Yoshiura K, Kanai Y, Ochiai A, Shimoyama Y, Sugimura T and Hirohashi S (1995) Silencing of the E-cadherin invasion suppressor gene by CpG methylation in human carcinomas. *Proc Natl Acad Sci USA* **92**: 7416–7419

© Cancer Research Campaign 1997